# Culture types and period impact gametophyte morphogenesis and sporophyte formation of eastern bracken

**DOI:** 10.1186/s13007-021-00786-7

**Published:** 2021-08-03

**Authors:** Bo-Kook Jang, Ju-Sung Cho, Shin-Ho Kang, Cheol Hee Lee

**Affiliations:** 1 Division of Animal, Horticultural and Food Sciences, Chungbuk National University, Cheongju, 28644 Republic of Korea; 2 Brain Korea 21 Center for Bio-Health Industry, Chungbuk National University, Cheongju, 28644 Republic of Korea; 3 Research Center for the Development of Advanced Horticultural Technology, Chungbuk National University, Cheongju, 28644 Republic of Korea; 4 Faculty of Bio-Pharmaceutical Industry, Semyung University, Jecheon, 27136 Republic of Korea

**Keywords:** Liquid suspension culture, Mechanical fragmentation, Phytohormones, *Pteridium aquilinum* var. *latiusculum* (Desv.) Underw. ex A. Heller

## Abstract

**Background:**

Liquid suspension culture efficiently proliferates plant cells and can be applied to ferns because it rapidly increases the fresh weight of gametophytes. This study assessed gametophyte proliferation and sporophyte production of *Pteridium aquilinum* var. *latiusculum* using a suspension culture method.

**Results:**

The growth curve linear phase of gametophyte cells was confirmed between 9 and 18 days of culture, and the subculture cycle was determined to be 2 weeks. A double-strength MS medium (fresh weight, 18.0 g) containing 2% sucrose and NH_4_^+^:NO_3_^−^ (120 mM, 40:80) was found to be the optimal liquid medium. Gametophytes obtained after suspension culture for 18 days did not normally form sporophytes in an ex vitro soil environment. However, this issue was resolved after changing the culture type or extending the culture period to 6 weeks. A short suspension culture period increased the fresh weight of fragmented and homogenized gametophytes but yielded numerous relatively immature gametophytes (globular forms of branching gametophytes, BG). Furthermore, differences in gametophyte morphogenesis and development were indicated by changes in endogenous phytohormone content. BG with immature development exhibited high accumulation of zeatin, jasmonic acid, and salicylic acid, and relatively low levels of abscisic acid and indole-3-acetic acid. The immature development of gametophytes directly affected sporophyte formation.

**Conclusions:**

This study maximized the advantages of liquid suspension culture using eastern bracken gametophytes and provides data to resolve any associated issues, thus facilitating efficient bracken production.

**Supplementary Information:**

The online version contains supplementary material available at 10.1186/s13007-021-00786-7.

## Background

Ferns are very popular horticultural plants worldwide [1]. They have a very high value as indoor/outdoor ornamental and landscaping plant materials. Ferns are rich sources of protein, essential amino acids, fatty acids, fiber, vitamins, and minerals [2], and they also occupy an important position as an edible plant [3, 4]. Among the ferns, eastern bracken (*Pteridium aquilinum* var. *latiusculum* (Desv.) Underw. ex A. Heller) is a popular wild vegetable in South Korea and its allies. Production of eastern bracken in South Korea, in particular, has reached 14,031 tons (72 million USD) [5], and it is cultivated as a high value commercial crop.

A liquid suspension culture of plant cells consists of cells and cell clumps dispersed and grown in an agitated medium [6]. The number of cells in the medium increases rapidly and approaches the maximum cell density [7]. Simultaneously, as mineral salts and carbohydrates in the medium are rapidly consumed as an energy source, the selection of medium components is important. In addition, liquid culture can resolve numerous issues associated with solid cultures, such as gaseous exchange and the gradients of nutrients in the medium. Suspension culture facilitates large-scale production of various plant species and is suitable for strategically producing high amounts of useful components (e.g., functional substances, natural products, and secondary metabolites) [8]. Furthermore, suspension culture can effectively preserve and propagate species that are difficult to cultivate and propagate, such as endangered wild plants. In suspension culture, callus and green globular bodies (ferns) derived from cells of numerous plant species have been used [8]. For example, *Pteris vittata* [9], *Nephrolepis exaltata* [10], *Drynaria quercifolia* [11], and *Platycerium bifurcatum* [12] were produced in suspension culture. However, ferns are easy to produce using gametophyte instead of callus [13, 14]. The gametophyte has a high regenerative ability, and this method can be maximized in liquid suspension cultures. However, the characteristics and development of gametophytes obtained by culture may potentially differ from those of gametophytes grown naturally depending on the culture type, such as solid, liquid, and airlift fermenter [15, 16]. Furthermore, exogenous gibberellic acid_4+7_ affects the regulation of gametophyte morphogenesis [17], and 6-Benzylaminopurine induces male development of homogenized gametophytes [18]. As such, gametophytes are sensitive to the culture type and phytohormone, and morphogenesis and endogenous phytohormone content potentially differ in accordance with gametophyte development and maturity [19–21].

This study proposed the mass production of eastern bracken gametophytes and sporophytes of high commercial value using liquid suspension culture. Furthermore, gametophyte morphogenesis and endogenous phytohormone content were investigated in accordance with culture type and culture period.

## Results

### Gametophyte growth in suspension culture

**Fig. 1:**
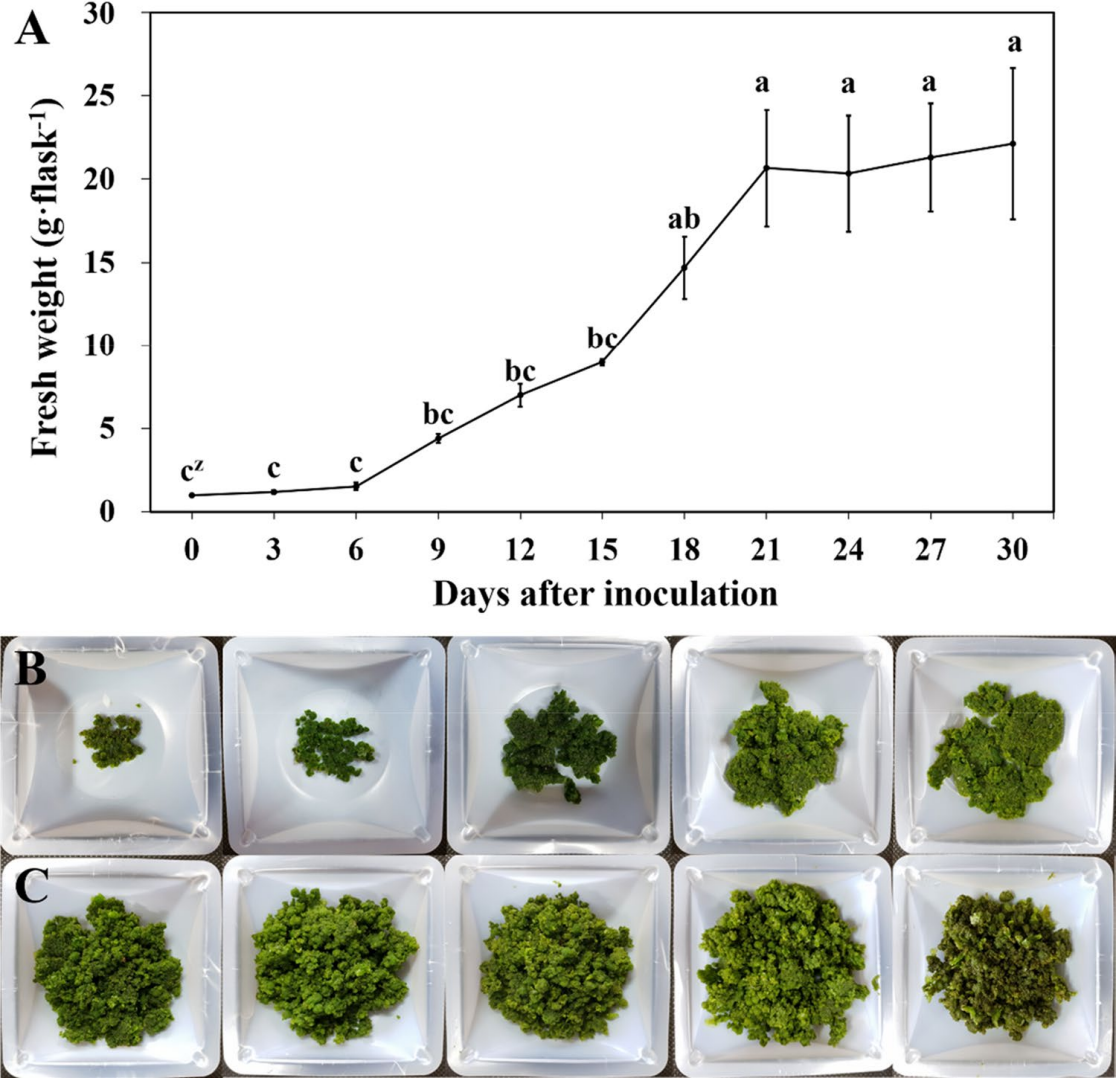
Suspension culture growth curve and fresh weight of eastern bracken gametophyte measured over 30 days. **A** Growth curve; **B**, **C** gametophytes in accordance with the liquid culture period (left to right: **B** 3, 6, 9, 12, 15, **C** 18, 21, 24, 27, and 30 days after inoculation). Vertical bars represent the mean ± standard error values (n = 4). ^z^Different letters indicate a significant difference using Duncan’s multiple range test at *P* < 0.05

Based on the growth curve for 30 days (Fig. 1A), the gametophytes increased rapidly from the 6th day (Fig. 1B). The fresh weight of gametophytes increased rapidly between 9 and 18 days and were 4.4, 7.0, 9.0, and 14.7 g. From the 18th day, browning and senescence were observed in some gametophyte cells (Fig. 1C).

### Conditions of gametophyte proliferation in suspension culture

**Fig. 2:**
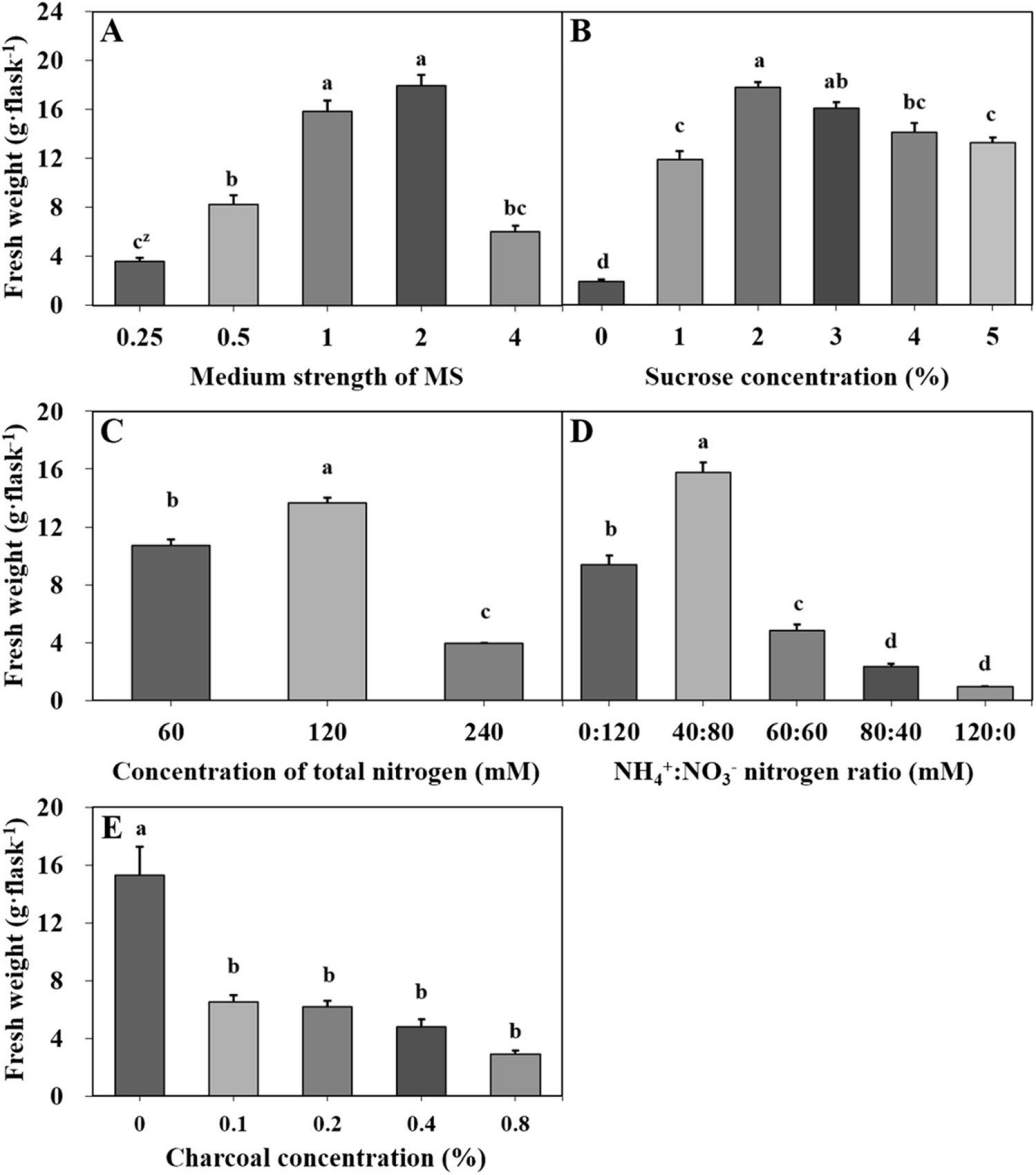
Effect of medium components on the fresh weight of eastern bracken gametophytes cultured for 18 days. **A** Culture media; **B** sucrose concentration; **C** total nitrogen concentration; **D** NH_4_^+^:NO_3_^−^ nitrogen ratio; **E** charcoal concentration. Vertical bars represent the mean ± standard error values (n = 4). ^z^Different letters indicate a significant difference using Duncan’s multiple range test at *P* < 0.05

The fresh weight of gametophytes (15.9 and 18.0 g) increased the most in 1 and 2MS medium (Fig. 2A). Fresh weight was most investigated in 2MS medium containing 2–3% sucrose (Fig. 2B). Regardless of the total concentration and ratio, the fresh weight of the gametophytes peaked in the medium with the nitrogen source adjusted to 120 mM NH_4_^+^:NO_3_^−^ (40:80) (Fig. 2C, D). Activated charcoal was not effective in increasing the fresh weight of gametophytes (Fig. 2E).

### Ex vitro sporophyte propagation in accordance with culture types

**Fig. 3:**
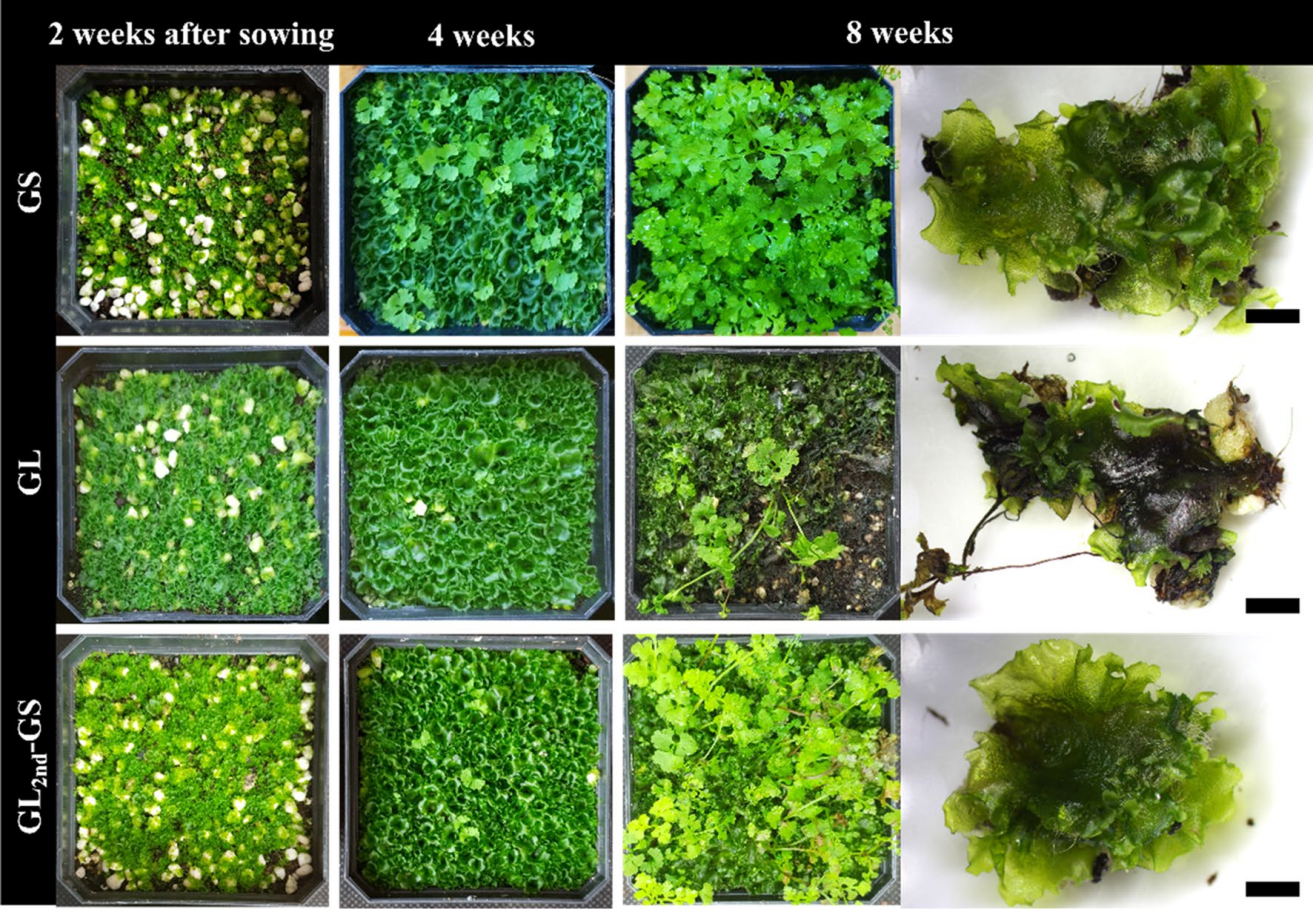
Development of eastern bracken gametophyte and sporophyte sown in accordance with the culture types in ex vitro conditions. GS, gametophyte fragmentation cultured for 6 weeks in solid medium; GL, gametophyte fragmentation cultured for 2 weeks in liquid medium; GL_2nd_-GS, liquid cultured gametophytes were subcultured twice at intervals of 2 weeks and then solid cultured for 6 weeks. Scale bars are 3 mm

GS produced 156.7 sporophytes per pot, whereas GL, GL_1st_, and GL_2nd_ hardly formed sporophytes per pot) (Table 1). However, GL_2nd_-GS produced 77.8 sporophytes per pot. Two weeks after sowing, all fragmented and homogenized gametophytes were successfully covered in ex vitro soil. Sporophyte formation occurred 4 weeks after sowing (Fig. 3). Nevertheless, unlike solid culture gametophytes, GL did not develop into sporophytes.

**Table 1:** Effects of different culture types on sporophyte formation and growth of eastern bracken in ex vitro conditions

Culture types	No. of sporophytes per pot	No. of leaves per plant	Leaf length (mm)	No. of roots per plant	Root length (mm)	Shoot FW (mg· plant^−1^)	Root FW (mg· plant^−1^)
GS	156.7 ± 16.50 a^z^	3.2 ± 0.29 b	26.4 ± 0.02 b	4.0 ± 0.00 ab	23.2 ± 1.07 a	11.6 ± 0.60 b	2.0 ± 0.19 a
GL	0.8 ± 0.48 b	3.7 ab	38.9 a	5.0 a	20.7 a	67.0 a	7.4 a
GL_1st_	8.3 ± 4.27 c	3.1 ± 0.22 b	23.3 ± 3.12 b	3.2 ± 0.44 b	22.5 ± 1.75 a	16.5 ± 5.33 b	5.8 ± 2.62 a
GL_2nd_	8.8 ± 3.09 c	3.5 ± 0.14 b	26.4 ± 0.91 b	4.0 ± 0.00 ab	20.9 ± 1.23 a	19.0 ± 2.66 b	7.3 ± 1.68 a
GL_2nd_-GS	77.8 ± 5.92 c	4.4 ± 0.22 a	28.7 ± 3.71 b	4.1 ± 0.22 ab	17.4 ± 2.37 a	28.6 ± 7.76 b	2.9 ± 1.07 a

GS, gametophyte fragmentation cultured for 6 weeks in solid medium; GL, gametophyte fragmentation cultured for 2 weeks in liquid medium; GL_1st_, liquid cultured gametophytes (GL) were subcultured and then liquid cultured for 2 weeks; GL_2nd_, liquid cultured gametophytes (GL_1st_) were subcultured and then liquid cultured for 2 weeks; GL_2nd_-GS, liquid cultured gametophytes (GL_2nd_) were subcultured and then solid cultured for 6 weeks

^z^Different letters indicate a significant difference using Duncan’s multiple range test at *P* < 0.05. FW, fresh weight

### Gametophyte morphogenesis in accordance with culture types

**Fig. 4:**
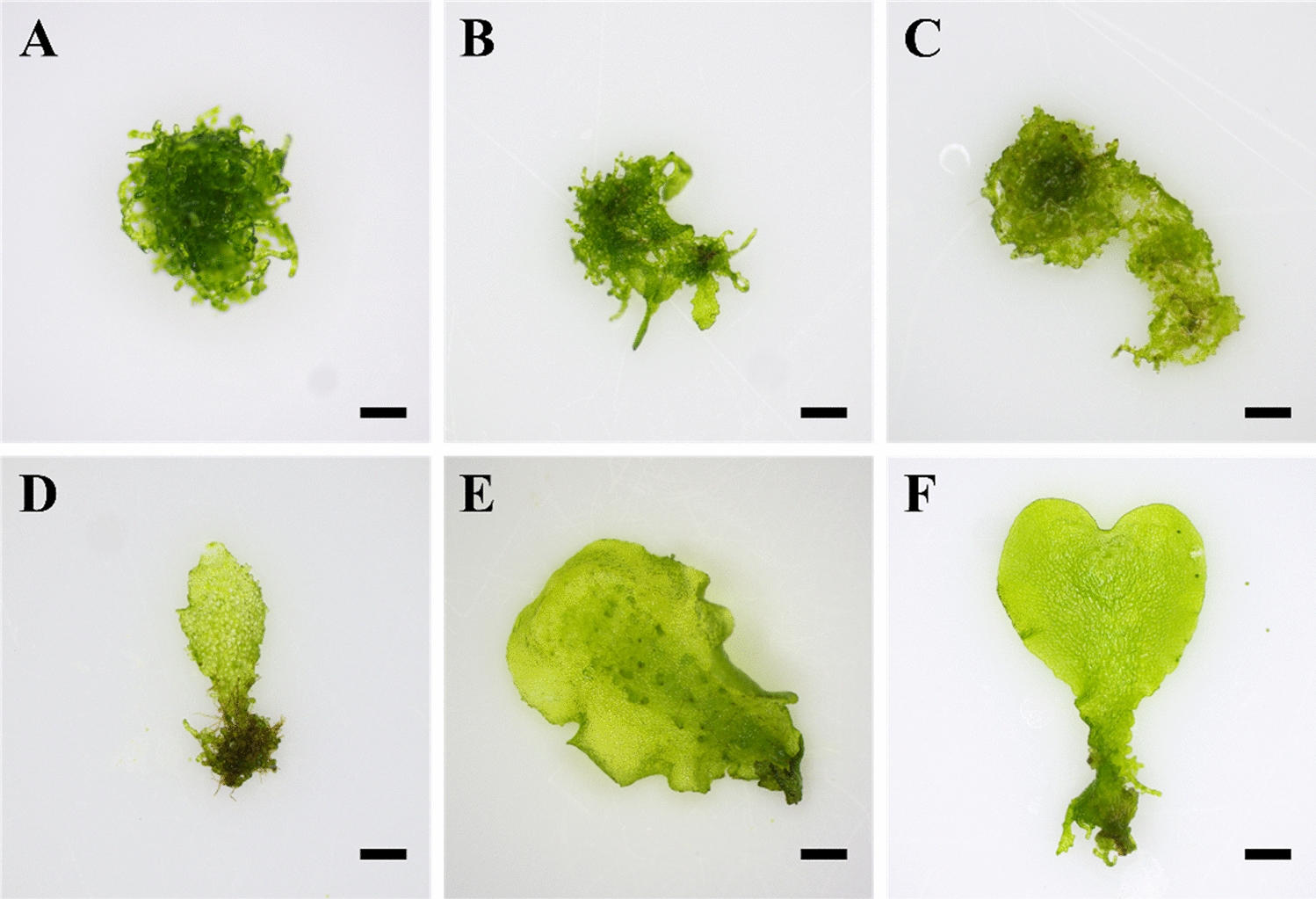
Regeneration and morphology of eastern bracken gametophytes cultured in liquid medium. **A** Globular forms of branching gametophyte; **B** and **C** branching gametophyte; **D** spatula shaped; **E** and **F** spatula-heart shaped. Scale bars are 1 mm

The morphogenesis of gametophytes obtained with different culture types was investigated (Table 2). GS mostly exhibited spatula gametophyte (SG) (89.7%) and spatula-heart gametophyte (S-HG) (10.3%). GL had a high proportion of branching gametophyte (BG) with immature morphogenesis (Fig. 4). For GL, GL_1st_, and GL_2nd_, the proportion of BG was high, from 54.5 to 82.4%. GL_2nd_-GS displayed a similar tendency to GS, with recovery of morphogenesis of SG (77.2%) and S-HG (22.8%). The GL series inhibited sporophyte formation regardless of morphogenesis (Additional file 1: Table S1).

**Table 2:** Effects of different culture types on gametophyte morphogenesis of eastern bracken

Culture types	Morphogenesis of regenerated gametophytes (%)			Total fresh weight (g)
	BG	SG	S-HG	
GS	0.0 c^z^	89.7 ± 2.30 a	10.3 ± 2.30 a-c	6.5 ± 0.32 a
GL	54.5 ± 9.58 b	17.7 ± 2.03 b	27.8 ± 9.03 a	6.9 ± 0.89 a
GL_1st_	82.4 ± 2.47 a	12.2 ± 2.88 b	5.4 ± 0.87 bc	5.2 ± 0.53 ab
GL_2nd_	79.5 ± 1.06 a	20.5 ± 1.06 b	0.0 c	5.5 ± 0.58 ab
GL_2nd_-GS	0.0 c	77.2 ± 9.77 a	22.8 ± 9.77 ab	4.5 ± 0.15 b

GS, gametophyte fragmentation cultured for 6 weeks in solid medium; GL, gametophyte fragmentation cultured for 2 weeks in liquid medium; GL_1st_, liquid cultured gametophytes (GL) were subcultured and then liquid cultured for 2 weeks; GL_2nd_, liquid cultured gametophytes (GL_1st_) were subcultured then liquid cultured for 2 weeks; GL_2nd_-GS, liquid cultured gametophytes (GL_2nd_) were subcultured and then solid cultured for 6 weeks; BG, globular forms of branching gametophyte; SG, spatula gametophyte; S-HG, spatula-heart gametophyte

^z^Different letters indicate a significant difference using Duncan’s multiple range test at *P* < 0.05

### Gametophyte endogenous phytohormone content according to culture types

Low levels of both ABA and IAA were detected in GL (1.0 and 16.9 ng· g^−1^) (Table 3). In contrast, high levels of zeatin, JA, and SA were detected in GL (109.4, 16.4, and 241.7 ng· g^−1^). However, GA_3_ content was not detected.

**Table 3:** Content of endogenous phytohormones in accordance with the culture type of eastern bracken gametophytes

Culture types	Endogenous phytohormone contents (ng· g^−1^)				
	ABA	IAA	Zeatin	JA	SA
Control	2.6 ± 0.06 a^z^	40.2 ± 1.55 a	103.5 ± 2.12 b	7.4 ± 0.30 b	112.2 ± 2.46 b
GS	2.4 ± 0.10 a	27.8 ± 1.61 b	77.6 ± 1.25 c	5.8 ± 0.48 c	46.7 ± 0.70 c
GL	1.0 ± 0.04 b	16.9 ± 0.31 c	109.4 ± 1.75 a	16.4 ± 0.12 a	241.7 ± 2.49 a

Control, spore-derived gametophytes cultured for 6 weeks in solid medium; GS, gametophyte fragmentation cultured for 6 weeks in solid medium; GL, gametophyte fragmentation cultured for 2 weeks in liquid medium

^z^Different letters indicate a significant difference using Duncan’s multiple range test at *P* < 0.05

### Gametophyte morphogenesis and ex vitro sporophyte propagation according to the culture period and density

As the culture period increased, the BG decreased, and the SG and S-HG percentages increased (Table 4). Furthermore, as the density of gametophytes per flask decreased, BG decreased, and S-HG increased. The most drastic change was investigated at a density of 0.1 g: 2 weeks (BG, 82.3%; SG, 10.3%; S-HG, 7.4%), 4 weeks (BG, 46.4%; SG, 33.4%; S-HG, 20.2%), and 6 weeks (BG, 7.1%; SG, 0.7%; S-HG, 92.2%).

**Table 4:** Effects of different culture periods and gametophyte density on gametophyte morphogenesis of eastern bracken

Gametophyte liquid culture		Morphogenesis of regenerated gametophytes (%)		
Period (week)	Gametophyte density (g)	BG	SG	S-HG
2	0.1	82.3 ± 3.48 a^z^	10.3 ± 2.88 cd	7.4 ± 3.99 d
	0.3	74.4 ± 3.54 ab	17.4 ± 4.79 b–c	8.2 ± 3.05 d
	0.5	63.6 ± 7.04 a–c	27.7 ± 6.25 a–c	8.7 ± 2.03 d
	1.0	63.2 ± 6.81 a–c	27.8 ± 4.99 a–c	9.1 ± 3.89 d
4	0.1	46.4 ± 16.55 cd	33.4 ± 11.47 ab	20.2 ± 13.82 cd
	0.3	62.3 ± 7.25 a–c	27.6 ± 2.81 a–c	10.0 ± 5.77 d
	0.5	47.6 ± 8.92 b–d	37.1 ± 9.06 a	15.3 ± 2.64 cd
	1.0	49.1 ± 13.1 b–d	30.3 ± 7.97 ab	20.6 ± 5.47 cd
6	0.1	7.1 ± 1.73 e	0.7 ± 0.48 d	92.2 ± 2.19 a
	0.3	28.0 ± 3.72 de	0.0 d	72.0 ± 3.72 b
	0.5	27.4 ± 8.32 de	8.6 ± 4.69 cd	64.0 ± 5.20 b
	1.0	65.4 ± 6.60 a–c	6.4 ± 3.61 d	28.2 ± 4.26 c
Significance				
Period (A)		***	***	***
Density (B)		NS	NS	**
A × B		**	NS	***

BG, globular forms of branching gametophyte; SG, spatula gametophyte; S-HG, spatula-heart gametophyte. ^z^Different letters indicate a significant difference using Duncan’s multiple range test at *P* < 0.05. NS, not significant; **, and *** indicates significance at *P* < 0.01, and 0.001, respectively

As the suspension culture period was extended to 6 weeks, sporophyte formation was recovered (Table 5). Sporophytes cultured for 6 weeks formed 56.0–115.0 sporophytes per pot, yielding different results depending on the density. Sporophytes were formed the most in gametophytes cultured for 6 weeks at a density of 0.1 g, and the growth of the individuals was relatively good.

**Table 5:** Effects of different culture periods and gametophyte density on sporophyte formation and growth of eastern bracken in ex vitro conditions

Gametophyte liquid culture		No. of sporophytes per pot	No. of leaves per plant	Leaf length (mm)	No. of roots per plant	Root length (mm)	Shoot FW (mg· plant^−1^)	Root FW (mg· plant^−1^)
Period (week)	Gametophyte density (g)							
2	0.1	7.3 ± 2.96 d^z^	3.6 ± 0.29 a	21.3 ± 4.27 a–c	3.4 ± 0.62 a	20.3 ± 3.46 ab	14.0 ± 3.62 ab	2.1 ± 0.45 b
	0.3	4.0 ± 1.00 d	3.4 ± 0.68 a	31.6 ± 11.67 a	4.6 ± 1.52 a	30.4 ± 11.46 a	52.2 ± 37.55 a	9.3 ± 6.64 a
	0.5	6.0 ± 1.00 d	3.3 ± 0.22 a	24.6 ± 2.91 ab	3.3 ± 0.36 a	24.8 ± 5.00 a	19.7 ± 4.55 ab	4.8 ± 1.40 ab
	1.0	5.0 ± 1.53 d	3.4 ± 0.29 a	22.8 ± 6.79 ab	3.2 ± 0.91 a	19.2 ± 2.44 ab	20.3 ± 8.53 ab	4.4 ± 1.47 ab
4	0.1	2.7 ± 0.33 d	3.2 ± 0.59 a	29.1 ± 8.96 a	3.3 ± 1.45 a	20.5 ± 7.02 ab	26.3 ± 15.63 ab	2.0 ± 1.14 b
	0.3	1.0 ± 0.58 d	1.2 ± 0.60 b	9.2 ± 4.59 b-d	0.8 ± 0.44 b	7.9 ± 4.25 bc	2.8 ± 1.44 b	0.2 ± 0.11 b
	0.5	0.3 ± 0.33 d	0.7 ± 0.67 b	4.7 ± 4.73 cd	0.7 ± 0.67 b	3.3 ± 3.26 c	1.4 ± 1.39 b	0.1 ± 0.07 b
	1.0	0.3 ± 0.33 d	0.3 ± 0.33 b	3.9 ± 3.90 d	0.7 ± 0.67 b	2.1 ± 2.05 c	0.5 ± 0.46 b	0.2 ± 0.17 b
6	0.1	115.0 ± 5.20 a	4.2 ± 0.08 a	29.3 ± 0.97 a	4.2 ± 0.08 a	16.1 ± 2.95 a–c	25.4 ± 2.17 ab	2.5 ± 0.50 b
	0.3	85.7 ± 15.19 b	3.9 ± 0.30 a	24.1 ± 0.31 ab	3.4 ± 0.30 a	18.6 ± 0.95 ab	15.6 ± 1.64 ab	1.7 ± 0.22 b
	0.5	56.0 ± 3.61 c	4.1 ± 0.17 a	30.9 ± 1.17 a	4.3 ± 0.14 a	23.6 ± 2.06 ab	28.9 ± 1.15 ab	4.4 ± 0.93 ab
	1.0	67.7 ± 3.18 c	3.7 ± 0.17 a	23.2 ± 1.82 ab	3.5 ± 0.14 a	20.4 ± 1.13 ab	13.0 ± 0.92 ab	1.6 ± 0.13 b
Significance								
Period (A)		***	***	***	***	***	NS	*
Density (B)		***	*	NS	NS	NS	NS	NS
A × B		***	*	NS	NS	NS	NS	NS

^z^Different letters indicate a significant difference using Duncan’s multiple range test at *P* < 0.05. NS, not significant; *, **, and *** indicates significance at *P* < 0.05, 0.01, and 0.001, respectively. FW, fresh weight

## Discussion

A suitable subculture period is essential for long-term preservation or proliferation of plant cells [1, 22]. Prompt depletion of nutrients in the suspension medium occurs with rapid growth of the cells [23]. Cell growth in liquid suspension culture is characterized by a lag phase, exponential phase, linear phase, deceleration phase, and a stationary phase [7]. Furthermore, eastern bracken gametophyte cells undergo the same growth process. The growth curve of gametophyte cells revealed that the gametophytes entered the exponential phase from the 6th day of culture. The culture period between 9 and 18 days revealed linear growth, and the deceleration phase was estimated to occur after 18 days. The fresh weight of the gametophytes stagnated from the 21st day and entered the stationary phase. Therefore, it was determined that the subculture period occurred before the deceleration phase (2 weeks post-culture).

A medium is the most important factor for plant growth in in vitro culture and contains various components for explant growth. It contains nutrients such as mineral salts, organic matter, and carbohydrates, and often contains activated charcoal, agar, and plant growth regulators. Among these, mineral salts and carbohydrates are indispensable for explant growth and proliferation [22, 23]. In this study, the difference in fresh weight of eastern bracken gametophytes, in accordance with the medium and components, was clearly revealed. Quarter- and half-strength MS liquid medium resulted in severe nutrient depletion, and quadruple-strength MS decreased the fresh weight owing to an excessive nutrient supply. The fresh weight of gametophytes depended on sucrose and nitrogen sources. However, activated charcoal did not contribute to this process. Through successive experiments, the liquid medium suitable for eastern bracken gametophyte proliferation was found to be a double-strength MS medium containing 2% sucrose and NH_4_^+^:NO_3_^−^; 120 mM (40:80). The eastern bracken gametophytes required very rich nutrients and components. Moreover, this has been reported in studies on *Equisetum arvense* [24], *Dicksonia sellowiana* [25], *Pteris tripartita* [26], and *Lemmaphyllum microphyllum* [27].

Gametophytes produced through liquid culture for 18 days displayed a very high proliferation rate; nonetheless, unfortunately, sporophytes were not normally formed in ex vitro soil environments. This issue resulted from differences in culture type and culture period, and gametophyte morphogenesis and developmental issues were also considered. In general, gametophytes form filamentous-, spatula-, spatula-heart-, heart-, and ribbon-shaped gametophytes during development [28]. The mature gametophyte is close to heart-shaped, and early gametophyte cells, which are immature and actively dividing, were filamentous-shaped. This morphogenesis can be classified in accordance with the division and development of gametophyte cells. Sheffield et al. [16] reported that the characteristics of cultured gametophytes may differ from those of gametophytes grown under natural conditions depending on the culture method. Gametophytes obtained from the suspension culture (GL series) had more than 55% of globular forms of BG, which is similar to that of filamentous gametophytes. The percentage of BG rapidly increased with repeated subculturing, and sporophytes were hardly formed. Meanwhile, in GS used as an initial explant, no BG was obtained after culturing, and only S-HG and SG were identified. GL_2nd_-GS displayed similar morphology to GS. In both conditions, sporophytes were normally formed, suggesting that gametophyte morphogenesis and development are closely associated with sporophyte formation.

Gametophyte morphogenesis is closely associated with cell division and development and is characterized by differential endogenous phytohormone content. The development of gametophytes and sporophytes is regulated by various hormone systems [29]. The ABA content of GL was less than half of that of the control and GS, in which gametophytes were relatively immature (GL). As GL contains a large amount of BG, cell division is quite active. ABA accumulated more in the mature stage than in the early stage, where cell division was active. Exogenous ABA inhibited cell division and elongation of *Lygodium japonicum* gametophyte [30] and that of *Mohria caffrorum* protonema [31]. Notably, the endogenous IAA content displayed a similar trend to that of ABA. Kosakivska et al. [20] reported a similar balance between the endogenous ABA and IAA contents analyzed from *Polystichum aculeatum* gametophytes of the same morphogenesis stage. Although auxin is involved in the growth of gametophytes, it accumulates primarily in young sporophytes and promotes development [32]. The IAA content was the lowest in the GL with BG; however, the control and GS with S-HG and SG displayed relatively high IAA levels, suggesting that similar to ABA, auxins accumulate more in mature gametophytes than in early-stage gametophytes. Gibberellin or gibberellin-like hormones are involved in the formation of reproductive organs and sex determination in ferns [33, 34] and are produced during gametophyte development; nonetheless, only GA_3_ was analyzed among various gibberellins, and all types of gibberellins were not synthesized herein. In the *Asplenium nidus* gametophyte, only GA_9_ was detected in the analysis of GA_1_, GA_3_, GA_4_, GA_7_, GA_9_, and GA_20_ [19]. Endogenous gibberellin is expected to play an important role in gametophyte development and morphogenesis. Therefore, it is necessary to analyze gibberellins in the biosynthetic pathway in the future. Cytokinins (CKs) are involved in cell division and organogenesis, among which zeatin promotes gametophyte and rhizoid development [35]. CKs can be detected at all stages because they promote cell division and organogenesis for development into mature plants. Zeatin, dihydrozeatin, zeatin-riboside, and isopentenyladenine were detected at similar levels in both gametophytes and sporophytes [19]. Kosakivska et al. [20] reported zeatin glucoside and isopentenyladenin in both the SG and sporophyte stages. In this study, zeatin was detected in the control, GS, and GL. Furthermore, high levels of both JA and SA were detected in GL. Surprisingly, endogenous JA and SA contents opposite trends to ABA and IAA contents. Although JA and SA are not often-used hormones in ferns, they have been used in some studies. Camloh et al. [36] reported that JA promotes early gametophyte development. Grzyb et al*.* [37] reported that exogenous SA reduced the endogenous IAA content of *Cyathea delgadii* explants. These results suggest that endogenous JA and SA hormones may contribute to early cell division in gametophytes, and these hormones potentially interact with other phytohormones. Moreover, the concentration of endogenous phytohormones clearly plays an important role in hormone regulation. Phytohormone concentration and function exert different effects depending on the cell and hormone receptor site. Additionally, qualitative and quantitative differences in phytohormones occur between adjacent tissues of the same species. For example, differences in gibberellin content and metabolism have been detected when comparing *Pisum sativum* L. embryos and seed coats [38]. The differing response to phytohormones is referred to as tissue 'sensitivity' (or responsiveness) [39, 40]. It is difficult to accurately define or measure sensitivity. For example, an increase in the number of phytohormone receptors, decrease in phytohormone breakdown, reduction in metabolism leading to fewer conjugated or inactive forms, and a decrease in transport away from the active site could all be interpreted as an increase in tissue sensitivity to an exogenous phytohormone [40]. Therefore, further studies and samples are required in this regard. Unfortunately, the sensitivity of gametophytic cells was not considered in this study. This should be considered when analyzing endogenous phytohormone concentration in the future.

As described above, morphogenesis (cell division and development) and the endogenous phytohormone content of gametophytes differed according to the culture type. Consequently, immature gametophytes influenced the inhibition of sporophyte formation. Short-term liquid culture effectively increased the fresh weight; however, it did not promote the regeneration and development of fragmented gametophytes. As the liquid culture period was extended to 6 weeks, sporophyte formation resumed. Furthermore, morphogenesis was confirmed to be closely associated with gametophyte density. The same volume of medium had a different nutrient-use efficiency in accordance with the gametophyte density [6, 7], potentially leading to developmental or morphogenetic changes. In fact, at 0.1 g density, the ratio of immature BG decreased significantly, and sporophyte formation increased. Furthermore, gametophyte morphogenesis and the percentage of sporophyte formation displayed the same tendency and recovered upon 6 weeks of culture. The culture period and gametophyte density were associated with gametophyte morphogenesis and the number of sporophytes formed and displayed a highly significant correlation (*P* < 0.001) (Tables 4 and 5).

## Conclusions

In summary, this study provides data on how to maximize the advantages of liquid suspension culture and resolve the associated issues, thus facilitating the efficient production of eastern bracken seedlings. Liquid suspension culture has been shown to rapidly increase the fresh weight of gametophytes. However, gametophytes obtained through suspension culture produced relatively immature gametophytes, which adversely affected the formation of sporophytes. In our study, the immature development of gametophytes directly affected sporophyte formation. This issue was resolved as the gametophytes matured after changing the culture type or extending the culture period to 6 weeks. Furthermore, BG with immature development exhibited high accumulation of zeatin, JA, and SA, and relatively low levels of ABA and IAA. It has been found that the content of endogenous hormones varies according to the maturity of gametophytes. This suggests that plant hormones are deeply involved in gametophyte development.

## Methods

### Plant materials

Sporophylls of *Pteridium aquilinum* var. *latiusculum* (Desv.) Underw. ex A. Heller (eastern bracken) were collected in a greenhouse at Chungbuk National University, Cheongju, Korea in September 2017. The sporophylls were dried in a paper box at 25 °C for 1 week, and spores and impurities were filtered out using a 100 μm sieve (Chunggye Sieve, Gunpo, Korea).

Spores were surface-sterilized and germinated in accordance with the methods of Jang et al. [41]. The spore solution (30 mg· 15 mL^−1^) was centrifuged (3 min, 1811×*g*), and the supernatant was discarded. The spores were sterilized with 1.4% (v/v) sodium hypochlorite (4% NaClO; Yuhanclorox Co., Ltd., Hwaseong, Korea) for 13 min and washed thrice with sterilized water. Finally, the spore solution was diluted to 1 mg· mL^−1^ of sterilized water and then inoculated in Knop medium [42]; the spores germinated at 25 °C under a 16/8 h photoperiod, with a light intensity of 30 μmol· m^−2^ ·s^−1^ photosynthetic photon flux density (PPFD). Gametophytes obtained from the spores were subcultured using double-strength MS [43] medium at eight-week intervals and then used for further experiments. The gametophyte material was transferred to the same medium without homogenization during subculture.

### Liquid suspension culture and gametophyte growth curve

The growth curves of eastern bracken gametophytes were assessed under suspension culture conditions. The culture was supplemented with 100 mL double-strength MS liquid medium [3% (w/v) sucrose, pH 5.8] in a 250 mL Erlenmeyer flask and agitated (VS-203D, Vision Scientific Co., Ltd, Daejeon, Korea) at 125 rpm. Gametophytes (1 g) were chopped using a scalpel and then suspension-cultured (1 g· 100 mL^−1^) [41]. The gametophytes were incubated for 30 days, and the fresh weight was determined at 3-day intervals. Thereafter, all cultures were carried out using this method. All suspension cultures were maintained at 25 °C, under a light intensity of 30 μmol· m^−2^ ·s^−1^, and a 16/8 h photoperiod, unless stated otherwise.

To determine the optimal liquid media for gametophyte proliferation, gametophyte fragments were cultured on quarter-, half-, full-, double-, and quadruple-strength MS liquid medium. Thereafter, the selected optimal medium (Fig. 2A, double-strength MS liquid medium) was controlled with various sucrose concentrations (0–5%, w/v), total nitrogen sources (60, 120, and 240 mM), nitrogen ratios (0:120, 40:80, 60:60, 80:40, and 120:0 mM), and activated charcoal (0%–0.8%, w/v) to assess gametophyte proliferation. NH_4_Cl (CAS 12125–02-9, Samchun Chemicals, Pyeongtaek, Korea) and KNO_3_ (CAS 7757–79-1, Wako Pure Chemical Industries, Ltd., Osaka, Japan) were used as nitrogen sources. The fresh weight of gametophytes was measured after 18 days. The solid medium was prepared with double-strength MS containing 2% sucrose, NH_4_^+^:NO_3_^−^, 120 mM (40:80), and 0.8% (w/v) agar (pH 5.8). The solid culture was sowed so that the ratio of gametophyte to medium was 0.3 g· 30 mL^−1^ and then cultured for 6 weeks.

### Responses of ex vitro sporophyte propagation in accordance with culture types

For ex vitro sporophyte propagation, the solid or liquid culture-derived gametophytes were fragmented and homogenized with a hand blender with 25 mL distilled water [27]. Gametophyte (1 g) was fragmented to various culture types and sowed under ex vitro soil conditions. The culture types were as follows (Additional file 1: Figure S1) fragmented gametophyte cultured for 6 weeks in solid medium (GS) and fragmented gametophyte cultured for 2 weeks in liquid medium (GL). Liquid cultured gametophytes were subcultured once or twice at 2-week intervals and then liquid cultured for 2 weeks (GL_1st_ or GL_2nd_). Liquid cultured gametophytes were subcultured twice at 2-week intervals and then solid cultured for 6 weeks (GL_2nd_-GS). Soil was mixed using sterilized horticultural substrate (Hanareum no. 2; Shinsung Mineral Co., Ltd., Goesan, Korea) and perlite (Newpershine no. 2; GFC. Co., Ltd., Hongseong, Korea) and filled in pots (75 × 75 × 75 mm). The soil volume was mixed at a 2:1 (v/v) ratio of horticultural substrate and perlite. Pots were placed in a plastic box, covered with a glass plate, and grown for 8 weeks. Water was sprayed on the gametophyte surface every 2-day 2 weeks after sowing. After 8 weeks, the growth of gametophytes and sporophytes was assessed. Thereafter, all ex vitro sporophyte propagations were carried out using the aforementioned method. All ex vitro conditions were maintained at 25 °C, under a light intensity of 43 μmol· m^−2^ ·s^−1^, 16/8 h photoperiod, and 85 ± 5% humidity, unless stated otherwise.

### Responses of gametophyte morphogenesis in accordance with culture types

Morphogenesis and fresh weight of gametophytes regenerated after culturing were assessed in accordance with the culture type. Regenerated gametophytes in each Erlenmeyer flask were washed with distilled water and enumerated, except for browning gametophytes. The total number of gametophytes was determined [A], and gametophyte morphogenesis (Fig. 4) was classified into three types [B]: globular forms of BG, SG, and S-HG. Morphogenesis (%) was determined as follows: [B – A] × 100%. Gametophytes, whose morphogenesis was classified in accordance with the culture type, were sowed ex vitro. However, morphogenesis types that were not regenerated were excluded from sowing.

### Change in gametophyte endogenous phytohormone content in accordance with the culture types

The endogenous phytohormone content in the gametophytes regenerated in accordance with the culture type was analyzed. As a control group, spore-derived gametophyte was analyzed. Gametophyte extraction from each treatment was conducted as indicated below. After freeze-drying, the homogenized 50 mg sample was placed in a 2 mL tube, and 500 μL extraction solvent (2-propanol/H_2_O/concentrated HCl (2:1:0.002, v/v/v, %) was added. The sample was homogenized at 100 rpm and 4 °C for 30 min. After agitation, 1 mL dichloromethane was added to each sample, agitated for 30 min (100 rpm, 4 °C), and each sample was centrifuged for 5 min (13,000 × *g*, 4 °C) (Smart R17 Plus; Hanil Scientific Inc., Gimpo, Korea). When the layers separated, 900 μL of the supernatant was transferred into a new 2 mL tube. The supernatant was evaporated using vacuum concentrators, and the recovered sample was finally dissolved in 100 μL methanol for subsequent use. The reference materials used were 2-cis,4-trans-ABA, GA_3_, IAA, JA, SA, and zeatin. They were dissolved in 1 mL methanol and diluted to 1 mg· mL^−1^. HPLC was performed using an Agilent 1260 series system (Agilent Technologies, Palo Alto, CA, USA). Chromatographic separation was achieved using an Agilent Eclipse Plus C_18_ column (4.6 × 50 mm^2^, 3.5 μm). The HPLC mobile phase was 0.1% formic acid in water (A) and 0.1% formic acid in methanol (B). The gradient was initiated at 5% (B), increased to 95% (B) for 1 min, and maintained at 95% (B) for 4 min. It was rapidly changed to 5% (B) for 0.1 min and retained for 0.9 min. The flow rate was set at 500 μL· min^−1^. The column oven temperature was set to 30 °C. The injection volume was 10 μL. The mass spectrometer was an API-4000 (AB Sciex, Concord, ON, Canada) equipped with an electrospray ionization source. Analysis was conducted in the negative mode and MRM mode. BioAnalyst version 1.6.1 and analyst software version 1.6.1 programs were used for equipment operation and data analysis, respectively. During ionization, high-purity nitrogen gas was used as the spray and drying gas. The gas pressure was set to 60 psi. Ion spray voltage (−4.5 kV) and an ionization source temperature of 600 °C were used. Q1 and Q3 were analyzed using LC–MS/MS in the multiple reaction monitoring mode with unit resolution. Three replicates were analyzed for each standard solution and sample.

### Responses of gametophyte morphogenesis and ex vitro sporophyte propagation in accordance with the culture period and density

Gametophyte morphogenesis and sporophyte formation were assessed in accordance with the liquid culture duration and gametophyte density. The culture duration (2, 4, and 6 weeks) and gametophyte density (fresh weight: 0.1, 0.3, 0.5, and 1.0 g per flask) were varied and evaluated after ex vitro seeding.

### Data collection and statistical analysis

Gametophyte morphogenesis was observed, and gametophytes were enumerated using a microscope (SZ61; Olympus, Tokyo, Japan). Gametophyte images were captured using a CMOS camera (eXcope F630; Dixi Sci., Daejeon, Korea) and eXcope 3.7.12277 software. In vitro gametophyte proliferation was evaluated using the fresh weight of cultured gametophytes (n = 4). To investigate the effect of sporophyte production, growth parameters of sporophytes were measured, including the number of sporophytes per pot, number of leaves, leaf length, number of roots, root length, and fresh weight. Triplicate sets were used to determine the levels of ex vitro sporophyte production, and sporophyte growth was investigated using 5 plants per replicate (n = 15). However, when <15 sporophytes were formed, all sporophytes were assessed. SAS version 9.4 (SAS Institute Inc., Cary, NC, USA) was used to determine the mean ± standard error values for each treatment, and factorial analysis was performed using Duncan’s multiple range test, with a significance level of *P* < 0.05. The culture period and gametophyte density data were analyzed using two-way ANOVA with SAS version 9.4.

## Supplementary Information

Additional file 1: **Table S1.** Effect of gametophyte morphogenesis in accordance with culture type on sporophyte formation and growth of eastern bracken in ex vitro conditions. **Figure S1.** Experimental design diagram of ex vitro sporophyte propagation according to culture types and periods. ^z^Liquid culture is more efficient for gametophyte proliferation than solid culture (shorter culture period and easier handling).

## Data Availability

Not applicable.
