# Supplementary material for: Culture types and period impact gametophyte morphogenesis and sporophyte formation of eastern bracken
Source: Plant Methods. 2021 Aug 3;17:87. doi: 10.1186/s13007-021-00786-7 (PMC8336368; doi:10.1186/s13007-021-00786-7)
Supplement: Supplementary file 1 — Additional file 1: Table S1. Effect of gametophyte morphogenesis in accordance with culture type on sporophyte formation and growth of eastern bracken in ex vitro conditions. Figure S1. Experimental design diagram of ex vitro sporophyte propagation according to culture types and periods. zLiquid culture is more efficient for gametophyte proliferation than solid culture (shorter culture period and easier handling). [file 13007_2021_786_MOESM1_ESM.docx]

**Additional file 1: Table S1.** Effect of gametophyte morphogenesis in accordance with culture type on sporophyte formation and growth of eastern bracken in ex vitro conditions.

| Culture types | Gametophyte morphogenesis | No. of sporophytes per pot | No. of leaves per plant | Leaf length (mm) | No. of roots per plant | Root length (mm) | Shoot FW  (mg·plant^−1^) | Root FW  (mg·plant^−1^) |
| --- | --- | --- | --- | --- | --- | --- | --- | --- |
| GL | BG | 6.8±3.12 | 3.8±0.23 | 26.3±4.08 | 4.5±0.66 | 22.5±2.03 | 17.7±7.05 | 2.4±0.86 |
|  | SG | 7.5±4.50 | 3.5±0.30 | 28.3±1.07 | 4.0±0.80 | 19.0±2.63 | 16.6±1.16 | 2.6±0.43 |
|  | S-HG | 6.8±2.69 | 3.9±0.07 | 34.9±1.12 | 4.8±0.08 | 27.7±2.50 | 32.6±3.35 | 5.1±0.95 |
| GL_1st_ | BG | 5.5±3.66 | 3.7±0.24 | 30.9±2.26 | 4.5±0.25 | 22.0±0.50 | 30.5±7.87 | 6.0±1.63 |
|  | SG | 0.0 | -^z^ | - | - | - | - | - |
|  | S-HG | 0.0 | - | - | - | - | - | - |
| GL_2nd_ | BG | 0.0 | - | - | - | - | - | - |
|  | SG | 9.3±2.93 | 3.8±0.14 | 23.0±0.56 | 4.1±0.22 | 19.5±0.74 | 19.2±0.89 | 7.3±0.27 |
|  | S-HG | - | - | - | - | - | - | - |
| GL_2nd_-GS | BG | - | - | - | - | - | - | - |
|  | SG | 53.3±7.51 | 4.0±0.31 | 26.6±2.31 | 3.5±0.07 | 14.3±1.57 | 16.8±2.09 | 1.2±0.18 |
|  | S-HG | 72.0±7.21 | 3.4±0.35 | 28.2±3.09 | 3.2±0.36 | 18.9±3.85 | 13.0±2.16 | 1.7±0.40 |

GS, gametophyte fragmentation cultured for 6 weeks in solid medium; GL, gametophyte fragmentation cultured for 2 weeks in liquid medium; GL_1st_, liquid cultured gametophytes (GL) were subcultured and then liquid cultured for 2 weeks; GL_2nd_, liquid cultured gametophytes (GL_1st_) were subcultured then liquid cultured for 2 weeks; GL_2nd_-GS, liquid cultured gametophytes (GL_2nd_) were subcultured and then solid cultured for 6 weeks; BG, globular forms of branching gametophyte; SG, spatula gametophyte; S-HG, spatula-heart gametophyte. ^Z^Not sown because the gametophyte was not regenerated. FW, fresh weight.


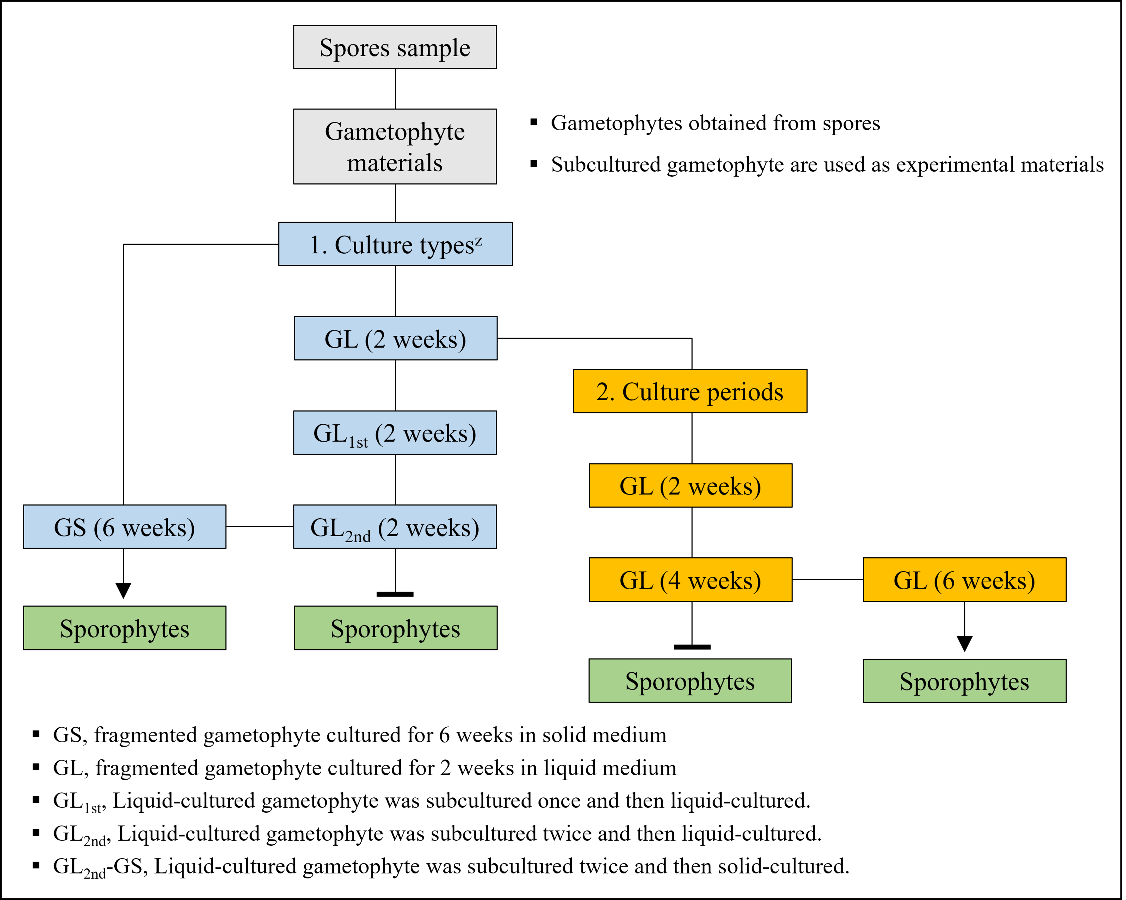
**Additional file 1: Figure S1.** Experimental design diagram of ex vitro sporophyte propagation according to culture types and periods. ^z^Liquid culture is more efficient for gametophyte proliferation than solid culture (shorter culture period and easier handling).
